# Mitochondrial Fitness Science Communication for Aging Adults: Prospective Formative Pilot Study

**DOI:** 10.2196/64437

**Published:** 2024-12-13

**Authors:** Cathy A Maxwell, Brandon Grubbs, Mary S Dietrich, Jeffrey T Boon, John Dunavan, Kelly J Knickerbocker, Maulik R Patel

**Affiliations:** 1 College of Nursing University of Utah Salt Lake City, UT United States; 2 Department of Health and Human Performance Middle Tennessee State University Murfreesboro, TN United States; 3 School of Nursing Vanderbilt University Nashville, TN United States; 4 Critical Care Medicine Vanderbilt University Medical Center Nashville, TN United States; 5 Peer-Tree Experiential Learning Franklin, TN United States

**Keywords:** older adults, physical activity, exercise, science communication, gerontology, usability, behavior change, mitochondria, fitness, health intervention, digital health, evaluation, feasibility study, community dwelling

## Abstract

**Background:**

A key driver that leads to age-associated decline and chronic disease is mitochondrial dysfunction. Our previous work revealed strong community interest in the concept of mitochondrial fitness, which led to the development of a video-based science communication intervention to prompt behavior change in adults aged 50 years and older.

**Objective:**

This study aimed to conduct formative and summative evaluations of MitoFit, an instructional, biologically based communication intervention aimed at improving physical activity in older adults aged 50 years and older.

**Methods:**

In the phase-1 formative evaluation, community-dwelling older adults (N=101) rated the acceptability, appropriateness, and helpfulness of our MitoFit video series, titled “How to Slow Down Aging Through Mitochondrial Fitness.” In the phase-2 summative evaluation, a subgroup of phase-1 participants (n=19) participated in a 1-month MitoFit intervention prototype to evaluate the intervention and data collection feasibility.

**Results:**

In phase 1, participants (mean age 67.8, SD 8.9 y; 75/100, 75% female) rated the MitoFit videos as acceptable (≥4 out of 5 on a Likert-scale survey; from 97/101, 96% to 100/101, 99%), appropriate (101/101, 100%), and helpful (from 95/101, 94% to 100/101, 99%) to support adaptation and continued work on our novel approach. Previous knowledge of mitochondria ranged from 52% (50/97; What are mitochondria?) to 80% (78/97; What are the primary functions of mitochondria?). In phase 2, participants (mean age 71.4, SD 7.9 y; 13/19, 72% female) scored better than the national average (50) on the Patient-Reported Outcomes Measurement Information System-19 for physical function (57), social activities (55.5), depression (41), fatigue (48.6), and sleep disturbance (49.6) but worse for anxiety (55.3) and pain interference (52.4). Additionally, 95% (18/19) of participants demonstrated MitoFit competencies within 2 attempts (obtaining pulse: 19/19, 100%; calculating maximum and zone 2 heart rate: 18/19, 95%; and demonstration of exercises: 19/19, 100%). At 1 month after instruction, 68% (13/19) had completed a self-initiated daily walking/exercise plan and submitted a daily activity log. A walking pulse was documented by 85% (11/13) of participants. The time needed to walk 1 mile ranged from 17.4 to 27.1 minutes. The number of miles walked in 1 month was documented by 62% (8/13) of participants and ranged from 10 miles to 31 miles. The number of days of strength training ranged from 2 to 31 days/month. Intervention feasibility scores ranged from 89% (17/19; seems easy to follow) to 95% (18/19; seems implementable, possible, and doable). Overall, 79% (15/19) stated an intention to continue the MitoFit intervention. Furthermore, 4 weeks after delivery of the prototype intervention, the percentage of participants doing aerobic activity for regular moderate activity increased from 35% (6/17) to 59% (10/17; *P*=.03).

**Conclusions:**

MitoFit was enthusiastically embraced and is a cost-effective, scalable, and potentially efficacious intervention to advance with community-dwelling older adults.

## Introduction

### Background

Key risk factors for noncommunicable chronic diseases (NCDs) are primarily lifestyle related, with physical inactivity as a leading risk [1]. Sedentary lifestyles contribute to the top 3 causes of NCD-related deaths: cardiovascular disease (CVD), type 2 diabetes, and cancer [2]. In the United States, only 1 in 4 adults meet national physical activity (PA) guidelines for aerobic and strength-training activity, with inactivity increasing as individuals age [3].

A growing body of literature identifies mitochondria as central biological hubs for impaired energy metabolism that leads to the development of NCDs [4,5]. Mitochondria are essential for the maintenance of cellular functions and homeostasis through the production of adenosine triphosphate (ATP) for cellular energy, as well as metabolites that support calcium regulation, redox balance, apoptosis, and protein synthesis [6,7]. Mitochondrial functions modulate multisystemic stress responses, with far-reaching implications for health and disease [8]. Mitochondrial dysfunctions influence all hallmarks of aging [9], which is a central focus of the rapidly accelerating field of geroscience. The centrality of mitochondria as a key factor for healthy aging [10] led to our teams’ interest in advancing understanding among the public at large, particularly middle-aged and older adults, as well as health care clinicians [11,12]. Our previous work revealed strong community interest in the topic of energy homeostasis, specifically, “how the body makes energy” [12], and we subsequently developed and tested a health and wellness program, AFRESH (Aging and Frailty: Resilience and Energy in the Second Half of Life), incorporating didactic content on mitochondrial fitness [13].

### This Study

To expand this work, we developed a video-based science communication intervention, MitoFit, aimed at prompting intrinsic motivation for exercise through enhanced science communication. We wished to explore whether lay-friendly delivery of information about the importance of mitochondria would lead to a change in behavior related to PA in aging adults. Intrinsic motivation entails doing an activity for its inherent meaning versus external results or rewards [14]. The reward of intrinsic motivation comes from the behavior itself and the psychological well-being associated with autonomy (need to be in charge) and competence (ability to do) [14]. Systematic reviews on barriers and motivators of PA in older adults report concerns about health and fitness and lack of motivation as key barriers [15]. Motivators of PA include a person-centered approach and inclusion of behavior capability principles [16]. Appreciation and respect for scientific information (vs nonscientific) also influence the uptake of health recommendations [17,18].

These concepts and principles guided the development of our MitoFit video series, titled “How to Slow Down Aging Through Mitochondrial Fitness,” as well as a prototype MitoFit intervention, a science-based communication intervention targeted to adults aged 50 years and older. This paper describes two phases of the MitoFit project: (1) formative evaluation to support the acceptability, appropriateness, and helpfulness of the MitoFit video series and (2) summative evaluation to pilot test the feasibility of a MitoFit prototype intervention. Our research questions were (1) Do community-dwelling adults aged 50 years and older find MitoFit videos acceptable, appropriate, and helpful? and (2) Can the MitoFit prototype intervention and data collection protocol be feasibly implemented with community-dwelling adults over a 1 month period? We hypothesized that the MitoFit videos would be acceptable (4 out of 5 on a Likert scale), that the MitoFit prototype intervention would be feasible (4 out of 5 on a Likert scale), and that our MitoFit approach using science communication and simple instructions would result in “self-initiation” of an exercise routine (≥70%) over a 1-month period.

## Methods

### Study Design

Development and testing of MitoFit were guided by the National Institutes of Health (NIH) Stage Model for Behavioral Intervention Development (more details in Multimedia Appendix 1) [19]. The model provides a development roadmap and advances best practices for generating, testing, and implementing interventions that can be delivered in real-world settings [20].

#### MitoFit Development: Phase 1, Stage 0

Stage 0 of the NIH Stage Model involves using the underlying basic science and relevant information for planning before intervention development [20]. During this stage, MitoFit videos were formulated within the domain of social cognitive theory (use of psychology, education, and communication) and protection motivation theory of persuasive communication to prompt individuals to engage in behavior to protect themselves [21,22]. The MitoFit content and intervention is guided by basic science on mitochondrial function and supported by expert consensus on the use of PA for health promotion and disease prevention [23]. Instruction about PA within MitoFit reflects principles of mitochondrial function, cardiorespiratory health (exercise and heart rate zones), and physical exertion levels that translate to improvements in physical fitness (increased physical endurance), and mitochondrial fitness (improved cellular oxygen consumption and VO_2_ max [maximal oxygen consumption]) [24,25].

#### MitoFit Development: Phase 1, Stage 1

Stage 1 of the NIH Stage Model comprises intervention generation and adaptation (Stage 1A) and pilot testing (Stage 1B) [20]. During this stage, we formed a team comprised of the principal investigator (CAM), a mitochondrial scientist (MP), a kinesiologist and exercise physiologist (BG), an experiential learning expert (JD), and graphic designers.

In Stage 1A, over a 1-year period (from January to December 2022), our team iteratively developed a series of 6 short videos (4-6 minutes each), titled “How to Slow Down Aging Through Mitochondrial Fitness.” Titles and content of individual videos (Textbox 1) were developed to convey a basic but simple and practical understanding of the importance of mitochondria for maintaining health and wellness. Our team was guided by literature on science communication to lay audiences [17,18]. Scripts were developed by the study team and subsequently incorporated into sequential storyboards (graphic organizers) with suggestions for graphics to accompany specific content. We were mindful of narratives that simplified content and captured attention through messaging that audiences would remember. Audio recordings to accompany graphic designs were produced in a recording studio with a spokesperson who is shown narrating the videos. Graphic designers combined audio recordings with graphics, animation, and sound to produce individual videos that the team reviewed, revised, and approved. Once the videos were developed, phase-1 testing was conducted (more details in the *Phase-1 Procedures* subsection below) to determine the acceptability, appropriateness, and helpfulness of the videos. After video development, a MitoFit intervention prototype was developed by the principal investigator (PI) and kinesiology researcher with specific expertise in exercise with older adults to accompany the video series (more details in Textbox 1).

**Titles of MitoFit videos and components of the MitoFit prototype intervention.**

**Video series: How to slow down aging through mitochondrial fitness**

- Video 1: Overview
- Video 2: What are mitochondria?
- Video 3: How mitochondria become damaged
- Video 4: How to create new and better mitochondria
- Video 5: Keeping our energy requirement in balance
- Video 6: Making mitochondrial fitness an essential part of daily life

**MitoFit intervention components**

- Calculation of maximum heart rate
- Determination of heart rate zone 2
- Completion of a 1 mile walk OR 2000 steps (continuous)
- Measurement of heart rate via pulse oximeter
- Demonstration of 5 strength training calisthenics:
  Chair stand
  Dumbbell shoulder press
  Wall push-ups
  Calf-raises
  Toe taps
- Provision of a 1-month tracking form to document the completed exercise

### Phase-1 Setting and Eligibility

Group sessions were scheduled at 5 sites (neighborhood: n=1; senior centers: n=3; and clinical research laboratory: n=1). Participants were recruited by posted flyers at each locale with the aim of obtaining a relatively large and diverse sample for qualitative feedback. Eligibility criteria included English-speaking, age of 50 years or older, ability to attend an in-person group session, and ability to walk independently. Eligible participants were offered a US $40 gift card upon completion of the group session. Phase-1 participants (N=101) completed written informed consent and were provided copies of the consent.

### Phase-1 Procedures

A total of 16 group sessions were scheduled at each of the 5 sites. Interested individuals either called the PI’s office and left a message or completed an electronic form to indicate interest in the study. The study project coordinator then contacted each person to confirm eligibility and schedule individuals for a group session of up to 12 participants per group. The sessions were led by the project coordinator (14 sessions) or another member of the study team (2 sessions). Each group session began with the completion of the informed consent, followed by the completion of a demographic form that also included 3 multiple-choice questions to assess baseline knowledge about mitochondria. The group leader then introduced the video series and provided participants with a pen and a blank sheet of paper to take notes. To prevent information, participants were instructed to hold their questions until all 6 videos were viewed and survey questions completed. Videos were viewed by the groups, 2 videos at a time, pausing in between for a focus group discussion. The focus groups were led by a group leader and included 4 open-ended questions with probes (more details in Multimedia Appendix 2).

The focus group discussions (3 per group session) were audio recorded, and the mp3 files were subsequently uploaded to a transcription service (Rev.com) and transcribed verbatim. Given the quantity and relevance of qualitative data from the focus groups, the authors developed a second publication that is under peer review in another journal. Upon completion of the focus groups, participants completed a 15-item survey on acceptability, appropriateness, and helpfulness (more details in the *Phase-1 Data Collection* subsection below). At the end of the survey, participants could provide their phone numbers to receive a call in 1 week for us to inquire about perceptions after participants had time to reflect more on the videos. After all data collection was completed, the PI offered to answer any questions that the participants had about the videos.

### Phase-1 Data Collection

Phase-1 participants (video viewers) completed a demographic form that included age, race or ethnicity, sex, marital status, education level, employment status, and 3 questions about previous knowledge of mitochondria. Questions for acceptability, appropriateness, and helpfulness were collected through surveys adapted from the Acceptability of Intervention Measure (AIM) and Intervention Appropriateness Measure (IAM), and responses reflected agreement (from completely disagree to completely agree) on a 5-point Likert scale [26]. Data were collected through paper surveys and double-entered and coded in REDCap (Research Electronic Data Capture; Vanderbilt University)—an encrypted, secure electronic data collection system [27,28].

### MitoFit Development: Phase 2, Stage 1B

During this summative phase of the NIH Stage Model, we assessed the feasibility of administering a prototype MitoFit intervention that would accompany the videos and the data collection processes planned for use with community-dwelling adults aged 50 years or older. The MitoFit intervention protocol entailed (1) viewing the MitoFit videos (simplified science communication on mitochondria), (2) didactic instruction about heart rate zones to create cellular demand for energy, (3) demonstration of 5 competencies (more details in Textbox 1), and (4) guidance on how to engage in a self-initiated walking and exercise plan. We proposed that our approach (science communication + simple instructions) would lead to self-initiation of a walking and strength training routine over a 1-month period. The age of 50 years was chosen for eligibility because our previous study revealed the aged 65 years or younger age group as having the strongest likelihood to change behavior [12]. For phase-2 pilot-testing of the prototype intervention, a subgroup of phase-1 participants volunteered (45/101, 45%), with a convenience sample of 19 attending 1 of 4 scheduled phase-2 sessions to receive the MitoFit prototype intervention. Testing of the video series and MitoFit intervention was conducted over 8 months (from May to December 2023).

### Phase-2 Prototype Development

Development of the MitoFit intervention prototype was based on evidence that individuals are more likely to self-initiate exercise routines that are simple, doable, and efficacious, with the ability to do the intervention within one’s own home or vicinity [29]. A central message of the intervention content is that “mitochondrial health and energy homeostasis is essential to healthy aging, and improved/maintained by creating the demand for energy through regular sustained physical activity.” The intervention is based on PA guidelines for Americans [30] and includes information about activities that comprise moderate and vigorous PA based on heart rate zones and physical exertion [31]. We specifically promoted moderate activity performed at heart rate zones 1 and 2 (50% to 70% of the maximum heart rate) [31,32].

### Phase-2 Eligibility

Participation in phase 2 was voluntary and offered to phase-1 participants on 4 dates at 3 sites. Eligibility included viewing of the MitoFit videos, the absence of heart failure, and the ability to walk independently for 20 minutes. The use of a cane or other walking aid was permitted. Participants were offered an additional US $40 gift card upon completion of the phase-2 group session.

### Phase-2 Procedures

After completion of a second informed consent, the phase-2 group sessions began with the completion of surveys for baseline PA and quality of life (more details in the *Phase-2 Data Collection* subsection below), followed by didactic instruction. Sessions were led by a certified exercise physiologist and kinesiology researcher (BG) to ensure the quality of intervention delivery and the safety of participants. Participants were instructed on heart rate zones and provided rationale and illustrations about how heart rate reflects use of nutrients (substrates: fatty acids or glucose) by mitochondria for fuel and energy. We promoted sustained and continuous PA in heart rate zones 1 and 2 for at least 20-30 minutes, emphasizing that these zones are typically perceived as doable at an exertion level in which participants can engage in conversation while walking. We also taught participants how to complete 5 basic calisthenic exercises, including chair stands, dumbbell shoulder presses, wall push-ups, calf raises, and toe taps. Participants were provided with a pulse oximeter (finger) and step counter (pedometer) that clipped to their clothing. Upon completion of didactic instruction, we explained how to estimate the age-based maximum heart rate and heart rate zone ranges for each participant using the following formula*: 207 – (0.7×age) = maximum heart rate* [32]. We then had participants demonstrate competencies: (1) calculation of their maximum heart rate, (2) determination of zones 1 and 2 heart rates, (3) obtaining their pulse by a pulse oximeter, and (4) completion of 5 calisthenic exercises. To conclude phase 2, participants were given a daily exercise tracking log and asked to record their initial walking pulse in heart rate zones 1 or 2, the number of minutes walked in 1 mile, the number of days of walking over 1 month, the number of miles walked each day, and the number of strength training sessions over 1 month. We told the participants that we would call them in 1 week to check in and inquire if they had initiated their personal plan and again in 1 month to ask them to send us a copy of their completed exercise tracking log.

### Phase-2 Data Collection

Phase-2 participants completed the Rapid Assessment of Physical Activity (RAPA; 9-items [7 itemsaerobic, 2 itemsstrength and flexibility]) [33] and the Patient-Reported Outcomes Measurement Information System-19 (PROMIS-19; 19 items) [34] that assesses the quality of life in 7 domains (physical function, anxiety, depression, fatigue, sleep, socialization, and pain). Upon completion of the group 2 session, participants completed the Feasibility of Intervention Measure (FIM) [26]. At 1 week, we called the participants to ask if they had self-initiated a walking and strength training plan and if they had used their pulse oximeter to monitor their heart rate when they walked. A month after the phase-2 group session, we called participants and readministered the RAPA, as well as the 3 questions about mitochondria that we had asked during the original phase-1 group session. We were interested if the participants retained knowledge about mitochondrial fitness. Finally, at 1 month after the phase-2 group session, we asked participants to send us (by email or text) a copy of their completed exercise tracking log. Collected data from paper sources were transferred into REDCap.

### Phase-1 and -2 Data Analysis

Data analysis was conducted using IBM SPSS Statistics (version 29.0). We used frequency distributions to summarize most of the data collected in both phases of the study. Mean (SD) were used for the continuous age distributions and median (IQR) were used for the PROMIS-19, RAPA, and pain ratings. Changes in the RAPA values (phase 2) were analyzed using Wilcoxon signed-ranks tests. Any interpretation of statistical significance used a critical alpha value of .05 (ie, *P*<.05).

### Ethical Considerations

The study received ethical approval from the Vanderbilt University Institutional Review Board (#230300). Recruitment occurred over 7 months from May 2023 to November 2023. Participants consented using paper consent forms, which were converted to electronic forms in REDCap. Participants received a US $40 check for study participation. Deidentified data are stored on protected and secured hard drives at Vanderbilt University. The study adhered to the STROBE (Strengthening the Reporting of Observational Studies in Epidemiology) guidelines for observational studies (Multimedia Appendix 3).

## Results

### Phase-1 Formative Evaluation

A total of 101 participants attended 1 of the 16 group sessions. A majority (74/101, 73.3%) was between the ages of 60 and 80 years, and 27% (27/101) identified as African American. Approximately half (53%, 53/101) had a college degree or higher, 75% (75/101) identified as female; almost half (47%, 48/101) were married or partnered, and 61% (62/101) were retired. Before viewing the videos, participants’ correct responses to the mitochondrial knowledge questions ranged from 52% (50/97; What are mitochondria?) to 80% (78/97; What is the primary function of mitochondria?; Table 1).

**Table 1:** Characteristics of study participants and previous knowledge of mitochondria (N=101).

Characteristics		Value
**Age (years; N=101), mean (SD; range)**		67.8 (8.9; 50-87)
**Age group (N=101), n (%)**		
	50-59	18 (18)
	60-69	40 (40)
	70-79	34 (34)
	80+	9 (9)
**Race (n=99), n (%)**		
	Asian	1 (1)
	African American	27 (27)
	White	71 (72)
**Sex (n=100), n (%)**		
	Female	75 (75)
	Male	25 (25)
**Marital or partnered status (n=101), n (%)**		
	Divorced or single	34 (34)
	Married or partnered	48 (48)
	Widowed	19 (19)
**Education level (n=100), n (%)**		
	High school or GED^a^	14 (14)
	Some college or associate degree	33 (33)
	Bachelor’s degree	29 (29)
	Master’s degree	13 (13)
	Doctoral degree	11 (11)
**Employment status (N=101), n (%)**		
	Full time	26 (26)
	Part time	9 (9)
	Unemployed	4 (4)
	Retired	62 (61)

^a^GED: General Educational Development.

### Acceptability, Appropriateness, and Helpfulness of Videos

Agreement scores (4 or 5 out of 5) for acceptability, appropriateness, and helpfulness ranged from 94% (95/101) to 100% (101/101), with the highest scores for appropriateness (Table 2). To explore potential intrinsic motivation related to the videos, we contacted 49 (48%) of the 101 phase-1 participants at 1 week who provided their phone numbers and asked, “Since you viewed the MitoFit videos, have you begun any physical activity that you were not already doing?” Of the 49 respondents, 35 (71%) stated that they had self-initiated walking or other exercise (eg, group class).

**Table 2:** MitoFit videos acceptability, appropriateness, helpfulness, and previous knowledge of mitochondria (N=101).

MitoFit video evaluation (Agree; N=101)^a,b^		Value, n (%)
**Acceptability**		
	Content engaging	100 (99)
	Length appropriate	97 (96)
	Information easy to understand	97 (96)
	Videos meet my approval	97 (96)
	Videos are appealing to me	98 (97)
	Like the videos	98 (97)
	Welcome the videos (n=99)	98 (99)
**Appropriateness**		
	Seem fitting for people aged >50 years	101 (100)
	Seem suitable for people aged >50 years	101 (100)
	Seem applicable for people aged >50 years (n=100)	100 (100)
	Seem like good match for people aged >50 years	101 (100)
**Helpfulness**		
	Increased my understanding of mitochondrial fitness	99 (98)
	Would be positive addition for older adults	100 (99)
	Motivated me to want to exercise more	95 (95)
	Feel more in control of my health and aging	95 (94)
**Self-initiated behavior change (1 week after phase 1;** **n=49)**		
	No	14 (29)
	Yes	35 (71)
**Previous knowledge of mitochondria (correct)**		
	What are mitochondria? (n=97)	50 (52)
	What is the primary function of mitochondria? (n=97)	78 (80)
	The best way to maintain mitochondrial fitness is to: (n=98)	59 (60)

^a^Unless noted otherwise below, the total number of respondents to the respective question was 101.

^b^A response of 4 or 5 on a Likert-response scale ranging from 1 to 5.

### Phase-2 Summative Evaluation

A total of 19 participants who confirmed that they met eligibility criteria volunteered to attend a phase-2 group session to receive the MitoFit prototype intervention. As shown in Table 3, their demographic characteristics were very similar to the entire group who participated in phase 1. Among 7 domains related to quality of life, median T-scores were better than the national average for US adults in physical function, depression, fatigue, sleep disturbance, and social activities and worse than average for anxiety and pain interference. The median pain rating was 2.0 (IQR 0-5) on the 0-10 scale.

**Table 3:** Characteristics of phase-2 MitoFit intervention participants (n=19).

Characteristic		Value
**Age (years; n=19), mean (SD; range)**		71.4 (7.9; 54-86)
**Age group (n=19), n (%)**		
	50-59	1 (5)
	60-69	5 (37)
	70-79	8 (42)
	80+	3 (16)
**Race (n=17), n (%)**		
	Asian	1 (6)
	African American	1 (6)
	White	15 (88)
**Sex (n=18), n (%)**		
	Female	13 (72)
	Male	5 (28)
**Marital or partnered status (n=19), n (%)**		
	Divorced or single	2 (11)
	Married or partnered	11 (58)
	Widowed	6 (32)
**Education level (n=19), n (%)**		
	High school or GED^a^	1 (5)
	Some college or associate degree	8 (42)
	Bachelor’s degree	7 (37)
	Master’s degree	3 (15.8)
	Doctoral degree	0 (0)
**Employment status (n=19), n (%)**		
	Full time	2 (11)
	Part time	1 (5)
	Retired	16 (84)
**PROMIS-19^b^ T-scores (n=19), median (IQR; range)**		
	Physical function (higher is better)	57.0 (42-57; 34-57)
	Participate in social activities (higher is better)	55.5 (50-65; 37-65)
	Anxiety (lower is better)	55.3 (40-58; 40-61)
	Depression (lower is better)	41.0 (41-56; 41-64)
	Fatigue (lower is better)	48.6 (33-54; 33-62)
	Sleep disturbance (lower is better)	49.6 (46-54; 32-59)
	Pain interference (lower is better)	52.4 (41-56; 41-67)
	Average rating of pain (0-10)	2.0 (0-5; 0-8)

^a^GED: General Educational Development.

^b^PROMIS-19: Patient-Reported Outcomes Measurement Information System-19.

### MitoFit Feasibility

The feasibility ratings in the “agree/completely” categories for the intervention prototype ranged from 89% (17/19; seems easy to follow) to 95% (18/19; implementable, possible, doable; Tables 4). Among the 4 competencies of the intervention protocol, all but 1 participant was able to demonstrate competence (Table 5). The same participant was unable to calculate the maximum heart rate and heart rate zones. Upon completion of intervention training, 18 (95%) of 19 participants agreed (score of 4 or 5) that the intervention was implementable, possible, and doable, and 17 (89%) agreed that the intervention was easy to follow.

At 1 month postintervention delivery, 15 (79%) out of 19 participants stated that they had implemented a walking and exercise plan, describing what they had done over the month. In total, 13 (68%) participants completed and submitted a self-initiated daily activity log (Table 6). Within the group of phase-2 participants, previous knowledge of mitochondria improved to 100% from their phase-1 responses for 2 of the 3 questions (primary function and how to maintain; Table 4).

Self-reports from the 19 participants in phase 2 indicated a statistically significant increase in aerobic and endurance scores on the RAPA surveys from baseline (phase 1) to 4 weeks after intervention (phase 2; *P*=.03). None of the participants achieved “active” status (30 minutes of moderate activity, 5 days per week). However, the percentage of participants in the “under active regular activities” increased from 35% (6/17) in phase 1 to 59% (10/17) in phase 2. While not statistically significant (*P*=.64), some improvement was noted in strength training, but not in flexibility.

**Table 4:** MitoFit prototype intervention feasibility and previous knowledge of mitochondria (n=19).

				Value, n (%)
**Feasibility**				
	**The intervention seems implementable.**			
		Disagree or completely disagree	1 (5)	
		Neither agree nor disagree	0 (0)	
		Agree or completely agree	18 (95)	
	**The intervention seems possible.**			
		Disagree or completely disagree	1 (5)	
		Neither agree nor disagree	0 (0)	
		Agree or completely agree	18 (95)	
	**The intervention seems doable.**			
		Disagree or completely disagree	1 (5)	
		Neither agree nor disagree	0 (0)	
		Agree or completely agree	18 (95)	
	**The intervention seems easy to follow.**			
		Disagree or completely disagree	1 (5)	
		Neither agree nor disagree	1 (5)	
		Agree or completely agree	17 (89)	
**Knowledge of mitochondria**				
	**What are mitochondria?**			
		Baseline, correct	14 (73)	
		4 Weeks after intervention, correct	12 (63)	
	**What is the primary function of mitochondria?**			
		Baseline, correct	18 (95)	
		4 Weeks after intervention, correct	19 (100)	
	**The best way to maintain mitochondrial fitness is to:**			
		Baseline, correct	10 (53)	
		4 Weeks after intervention, correct	19 (100)	
**Completed and submitted MitoFit tracking form**				13 (68)
**Intend to continue the MitoFit walking and exercise plan**				15 (79)

**Table 5:** Baseline physical activity (RAPA^a^) and demonstration of MitoFit competencies (n=19).

				Baseline, n (%)	4 Weeks after intervention, n (%)		*P* value	
**RAPA (n=17)**								
	**Aerobic**							.03
		Sedentary	1 (6)		0 (0)			
		Under-active	3 (18)		1 (6)			
		Under-active, regular light activities	7 (41)		6 (35)			
		Under-active, regular moderate activities	6 (35)		10 (59)			
		Active	0 (0)		0 (0)			
	**Strength and flexibility**							.64
		None	3 (18)		4 (24)			
		Strength	3 (18)		6 (35)			
		Flexibility	6 (35)		0 (0)			
		Both strength and flexibility	5 (29)		7 (41)			
**Demonstration of MitoFit competencies**								
	**Obtains pulse by pulse oximeter**							
		Unable to perform	—^b^		0 (0)			
		Yes, with more than 2 attempts	—		0 (0)			
		Yes, with 2 attempts	—		1 (5)			
		Yes, after first attempt	—		18 (95)			
	**Calculates maximum heart rate**							
		Unable to perform	—		1 (5)			
		Yes, with more than 2 attempts	—		0 (0)			
		Yes, with 2 attempts	—		2 (11)			
		Yes, after first attempt	—		16 (84)			
	**Calculates zone 2 heart rate**							
		Unable to perform	—		1 (5)			
		Yes, with more than 2 attempts	—		0 (0)			
		Yes, with 2 attempts	—		2 (11)			
		Yes, after first attempt	—		16 (84)			
	**Demonstrates 5 strength training exercises**							
		Unable to perform	—		0 (0)			
		Yes, with more than 2 attempts	—		0 (0)			
		Yes, with 2 attempts	—		2 (11)			
		Yes, after first attempt	—		17 (90)			

^a^RAPA: Rapid Assessment of Physical Activity.

^b^Not applicable.

**Table 6:** Summary of submitted tracking log data (by chronological age) at 1 month after the phase-2 group session with individual-reported information (n=13).

Participants	Age group (years)	Walking pulse (beats per minute)	Minutes per mile, n	Days walked, n	Miles per month, n	Days of strength training, n
ID 1	60-64	98	21.0	10	10	0
ID 2	60-64	92	23.0	20	20	10
ID 3	60-64	118	17.38	15	18	7
ID 4	65-69	98	25.0	10	10	8
ID 5	65-69	105	18.0	29	29	6
ID 6	65-69	98	23.3	11	16.5	2
ID 7	70-74	104	22.0	29	29	12
ID 8	70-74	120	27.08	25	ND^a^	6
ID 9	75-79	112	20.0	31	31	31
ID 10	75-79	128	20.16	31	ND	11
ID 11	75-79	—^a^	—	21	ND	0
ID 12	80-84	62	22.0	31	ND	10
ID 13	80-84	—	—	12	ND	0

^a^ND: Not documented.

^b^Not available.

Among the 19 intervention participants, 1 dropped out 1 week before starting a walking plan, and 1 could not be reached for the 1-month follow-up despite repeated attempts. We were able to contact 17 participants for follow-up at 1-month post intervention. Among all intervention participants, 15 (79%) out of 19 expressed a stated intention to continue a walking plan. A total of 13 (68%) participants submitted copies of their exercise tracking logs (JPEG or PDF files) either by email or by phone text. Four participants were contacted but did not submit their tracking logs. Furthermore, 2 of the 4 stated that they tried to submit but were unsure of how. From the 1-month follow-up phone calls, an example of a blank tracking log is provided (see Multimedia Appendix 4).

### Submitted Tracking Logs

Among the 13 participants who submitted their tracking logs, ages ranged from 64 to 81 years (Table 6). A total of 11 (85%) out of 13 used their pulse oximeter to obtain a walking pulse in zones 1 or 2 (range 62-120). All 13 submitters documented the number of minutes walked and the days (dates) walked. The mean number of days walked per month was 21.2 days, ranging from 10 to 31 days. Minutes walked per day ranged from 5 to 74 minutes. All 13 submitting participants documented the days (dates) that they did the strength training calisthenic exercises. In addition, 10 (77%) out of 13 engaged in strength training exercises, ranging from 2 to 31 days/month over the 1-month period. Furthermore, 2 participants did not engage in strength training exercises. At the bottom of the tracking form, participants could fill in weekly totals and 8 (62%) participants completed the section. One participant documented COVID-19 as the cause for 9 missed days. One participant documented the location of their daily walks (ie, senior center, treadmill, or neighborhood).

## Discussion

### Principal Findings

Formative and summative evaluation of MitoFit demonstrated the acceptability, appropriateness, and helpfulness of our video series on mitochondrial fitness targeted to adults aged 50 years and older. Feasibility of the MitoFit intervention prototype was demonstrated through FIM survey results, demonstration of competencies, self-initiation of personal walking plans (15/19, 79%), and submission of completed exercise tracking logs (13/19, 68%). Of note, we were only testing intervention feasibility and not efficacy or effectiveness. In addition, qualitative data from the phase-1 focus groups (reported in a separate publication) demonstrated evidence of cognitive restructuring (mental adjustments to previous unhelpful beliefs), influenced by science communication in the videos, didactic content, and illustrations. Comments from phase-2 participants during 1-month follow-up phone calls reflected a change in how individuals regard the importance of exercise for healthy aging. For example, participant #7 stated, “Thank you for the opportunity to be a part of something that has forever changed how I view life and health.” Another participant (#81) stated, “Thank you for doing this study! It has really motivated me to keep it up and it’s not too hard to do.”

From a public perspective, the development of the MitoFit videos and intervention prototype was prompted by our team’s awareness that rising rates of obesity and chronic disease in the United States constitute a public health crisis [35] and that strategies to address this crisis should be efficacious, cost-effective, and scalable to reach large numbers of people. Interventions to increase PA in adults abound in scholarly literature, highlighting high costs and difficulty in sustaining behavior change that will truly alter health outcomes. Azjen’s [36] Theory of Planned Behavior posits that beliefs provide the basis for intention to engage in specific behaviors. Science communication can influence beliefs and intentions [17,18]. The MitoFit videos and intervention were developed to prompt protection motivation by increasing knowledge about complex but important health messaging [37]—the importance of mitochondria as essential biological hubs that play a major role in metabolic health and energy homeostasis. We also desired that MitoFit content would impress participants with an understanding that a higher step count leads to better health [38,39], as well as a sense that daily walking and exercise could be simple, doable, and sustainable. We achieved our aims for this pilot work and will use study findings to support the expansion of MitoFit to larger audiences using cost-effective implementation methods (eg, learning platform, digital twin technology, and public health outreach).

Understanding the role of mitochondria in health and disease will likely continue to grow exponentially over the next decade [40]. Our team supports advancing understanding of mitochondrial fitness among health care providers, equipping them to engage in dialogue with patients to promote proactive decision-making and preventive medicine. Directing patients to lay-friendly sources of publicly available information on mitochondrial health could be pivotal in turning the tide of obesity and chronic disease in the United States and other countries. Geroscience is a new field of medicine that combines aging biology, chronic disease, and health to understand how geroscience-guided interventions, including behavioral interventions like MitoFit, affect the progression of chronic disease [41]. Mitochondrial health intersects with every hallmark of aging and is the foundation of metabolic programming at the cellular level. Aging is characterized by a shift from catabolism to anabolism and from cellular energy production to an emphasis on repair processes as tissue- and organ-level processes begin to breakdown. Explaining these complex mechanisms through scientific communication to both health care and lay audiences is imperative to address our health care challenges.

### Strengths and Limitations

Strengths of our study include our novel science communication approach for prompting behavior change, as well as a diverse sample of participants for assessing the acceptability of the MitoFit videos. Our study achieved promising results through a low-cost communication approach. Our cost-effective approach to intervention delivery and team involvement over the 1-month postintervention period could also be viewed as a limitation, since we had minimal engagement with participants during the 1-month post–phase-2 group session period. Our approach was intentional to enable assessment of whether participants would “self-initiate” behavior change based primarily on communication strategies to prompt planned behavior, a crucial component of motivation and behavior sustainability. We acknowledge participants’ expectation of 1-week and 1-month follow-up phone calls may have also contributed to motivation. Quantifying motivation for change would strengthen our approach in future studies. Testing the MitoFit intervention solely for feasibility and not efficacy could also be viewed as a limitation of the study.

Regarding the administration of questions about previous knowledge of mitochondria, participants completed 3 multiple-choice questions developed by the study PI. During phase 2, participants were readministered the questions and we reported a decrease in correct answers for #1 (What are mitochondria?) from 73% (14/19) to 63% (12/19). Of note, the 4 choices provided for answers could have been confusing and the time span between phase 1 and phase 2 (up to 2 weeks) could have caused participants to forget. Conversely, question #3 (the best way to maintain mitochondrial fitness) results showed an increase in knowledge from 53% (10/19) to 100% (19/19). Another potential limitation of the intervention prototype testing was a disproportionate percentage of adults aged 70 years or older (11/19, 58%), which could have contributed to greater physical limitations, as well as lower digital literacy as reflected in the inability to submit tracking logs by email or text. As adults age, they are less likely to engage in enough PA to cause mitochondrial adaptations. Future work will include a broader sample of age groups. Other limitations of the study include the omission of a posttest on mitochondrial knowledge with phase-1 participants, the short (1-month) follow-up period, and the omission of flexibility training instruction (a component of the RAPA) for phase-2 participants. Future studies with MitoFit should include a 1- or 2-year follow-up to determine true sustainability and attrition, as well as the inclusion of flexibility exercises.

### Conclusion

Mitochondrial health is a critical mediator of cellular function and metabolic programming that translates to overall health and wellness. These biological processes are complex. However, communication to lay audiences and health care providers will be paramount in the future to promote proactive and preventive behavior change. Communication strategies and interventions like MitoFit that promote PA (walking) are simple and doable, with the potential to induce beneficial mitochondrial adaptations that could translate to improvements in health and prevention of chronic disease in aging adults [41].

## Appendix

Multimedia Appendix 1 MitoFit development based on the NIH Stage Model: formative and summative phases. NIH: National Institutes of Health.

Multimedia Appendix 2 Phase-1 focus group qualitative questions.

Multimedia Appendix 3 The STROBE (Strengthening the reporting of observational studies in epidemiology) guidelines for observational studies.

Multimedia Appendix 4 Example of a blank tracking log.

## Abbreviations

AFRESH: Aging and Frailty: Resilience and Energy in the Second Half of Life
AIM: Acceptability of Intervention Measure
ATP: adenosine triphosphate
CVD: cardiovascular disease
FIM: Feasibility of Intervention Measure
NCD: noncommunicable chronic disease
NIH: National Institutes of Health
PA: physical activity
PI: principal investigator
PROMIS-19: Patient-Reported Outcomes Measurement Information System-19
RAPA: Rapid Assessment of Physical Activity
REDCap: Research Electronic Data Capture
STROBE: Strengthening the Reporting of Observational Studies in Epidemiology
VO2 max: maximal oxygen consumption

## Data Availability

The datasets and analyses generated during the study are available from the corresponding author upon reasonable request.
