# Supplementary material for: Mitochondrial Fitness Science Communication for Aging Adults: Prospective Formative Pilot Study
Source: JMIR Form Res. 2024 Dec 13;8:e64437. doi: 10.2196/64437 (PMC11681289; doi:10.2196/64437)
Supplement: Multimedia Appendix 1 [file formative_v8i1e64437_app1.docx]

**Multimedia Appendix 1.** MitoFit Development based on NIH Stage Model: Formative and Summative Phases

| NIH Stage Model for Behavioral Intervention Development | | | | | |
| --- | --- | --- | --- | --- | --- |
| Phase 1: Formative Evaluation | | | Phase 2: Summative Evaluation | | |
| Stage 0 | Stage 1A | | Stage 1B | | |
| Development | Procedures | Analysis | Procedures | Follow-up | Analysis |
| -Background research on mitochondrial function & cellular health  -Exploration of mechanism of change (protection motivation) through science communication  -Video-development team  -Script writing | -MitoFit video development with refinement by team of experts  -Video-viewing sessions with community-dwelling adults, followed by focus group discussions  N=101 | -Quantitative results of acceptability, appropriateness and helpfulness related to videos  -Thematic analysis of participant feedback | -MitoFit prototype instruction, led by exercise physiologist.  -Demonstration of competency by pilot study participants. | -Follow-up phone call at one week with pilot participants to assess extent of self-initiated behaviors.  -Return of completed MitoFit tracking logs by pilot participants. | -Analyze feasibility of conducting MitoFit intervention.  -Refine for further testing based on pilot study results. |
