# Supplementary material for: Mitochondrial Fitness Science Communication for Aging Adults: Prospective Formative Pilot Study
Source: JMIR Form Res. 2024 Dec 13;8:e64437. doi: 10.2196/64437 (PMC11681289; doi:10.2196/64437)
Supplement: Multimedia Appendix 2 [file formative_v8i1e64437_app2.docx]

**Multimedia Appendix 2**. Phase 1 Focus Group Qualitative Questions

Qualitative questions

- What is your initial reaction to the videos using one or two words?
- What do you think is the most important take-way from the videos?
  - What new information did you learn?
  - How did the videos help in understanding mitochondrial fitness?
- What changes, if any, would you suggest to make the videos more effective?
- What was the most memorable part of the videos for you?
  - How did the videos improve your understanding of mitochondrial fitness?
  - How could you use the information from the videos in your day-to-day life?
