# Supplementary figures and images for: Mitochondrial Fitness Science Communication for Aging Adults: Prospective Formative Pilot Study
Source: JMIR Form Res. 2024 Dec 13;8:e64437. doi: 10.2196/64437 (PMC11681289; doi:10.2196/64437)

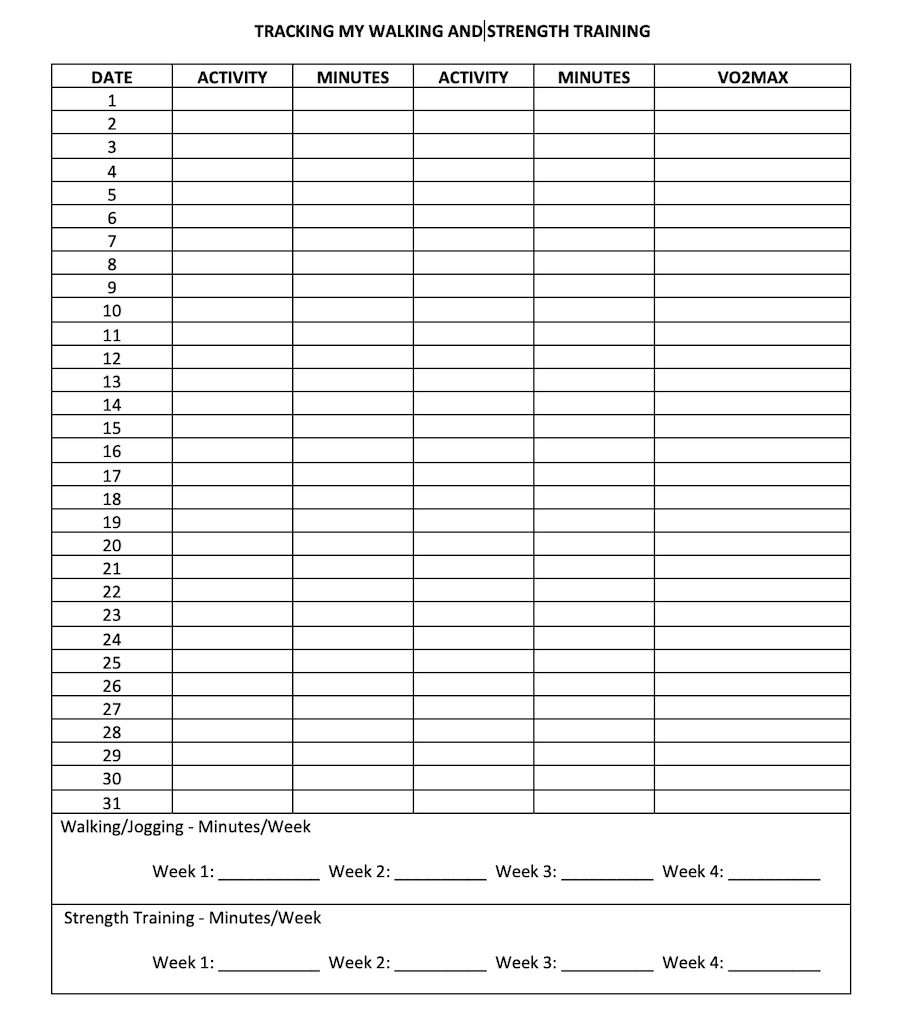

Supplement: Multimedia Appendix 4 [file formative_v8i1e64437_app4.png]
